# Depression and Endothelial Dysfunction in Psoriatic Arthritis: Is There Any Possible Relationship?

**DOI:** 10.3389/fmed.2021.669397

**Published:** 2021-08-27

**Authors:** Enrico De Lorenzis, Angela Di Giorgio, Gerlando Natalello, Antonio Nesci, Giacomo Tanti, Pietro Rubortone, Donatella Lucchetti, Maria Rosaria Magurano, Clara Di Mario, Barbara Tolusso, Giusy Peluso, Angelo Santoliquido, Elisa Gremese

**Affiliations:** ^1^Institute of Rheumatology, Catholic University of the Sacred Heart, Rome, Italy; ^2^PhD Program in Biomolecular Medicine - Cycle XXXV, University of Verona, Verona, Italy; ^3^Department of Internal Medicine, Angiology Unit, Catholic University of the Sacred Heart, Rome, Italy; ^4^Department of Translational Medicine and Surgery, Catholic University of the Sacred Heart, Rome, Italy; ^5^Unit of Clinical Psychology, Fondazione Policlinico Universitario A. Gemelli IRCCS, Rome, Italy; ^6^Division of Rheumatology, Fondazione Policlinico Universitario A. Gemelli IRCCS, Rome, Italy

**Keywords:** psoriatic arthritis, depression, flow-mediated dilatation, cardiovascular risk, interleukin-6, tumor necrosis factor-α, interleukin-17

## Abstract

**Background:** Cardiovascular events (CVEs) are the first cause of death in patients with psoriatic arthritis (PsA). Depression is a recognized risk factor in cardiovascular events and is frequently associated with PsA. Flow-mediated dilatation (FMD) is a widely used method for assessing endothelial dysfunction, a parameter with strong prognostic implications for CVEs. The study aims to explore the relationship between FMD, depressive symptoms and serum cytokines in a cohort of patients with PsA.

**Patients and Methods:** FMD was assessed in 50 consecutive PsA patients aged between 30 and 75 years without known cerebrovascular and coronary heart disease or diabetes. Depressive symptoms were reported using the related subscale of the Hospital Anxiety and Depression Scale (HDS). Disease features, activity indexes, and adjusted Framingham risk score (aFRS) were calculated. Serum level of IL-6, TNF-α, and IL-17A were also assessed.

**Results:** In PsA patients (age 50.7 ± 10.2 years, male 42%, disease duration 5.9 ± 3.3 years, Disease Activity in PSoriatic Arthritis (DAPSA) score 14.0 ± 9.4) FMD inversely correlated with the severity of depressive symptoms according to HDS (ρ = −0.339, *p* = 0.016), age (ρ = −0.507, *p* = 0.001), aFRS (rs = −0.453, *p* < 0.001), duration of PsA (ρ = −0.507, *p* = 0.001), intensity of pain (ρ = −0.507, *p* = 0.001), and DAPSA (ρ = −0.507, *p* = 0.001). No statistically significant correlation was found between FMD or HDS and serum cytokines concentrations. HDS predicted FMD in a model adjusted for age, aFRS, PsA duration, and pain intensity (β = −0.271, *p* = 0.008), with depressive symptoms contributing directly to 6.4% of the variance.

**Conclusions:** Depressive symptoms correlate with endothelial dysfunction with an exposure-response pattern in our cohort of PsA patients.

## Introduction

Cardiovascular events (CVEs) are the leading cause of death in patients affected by psoriatic arthritis (PsA), who indeed present an increased rate of myocardial infarction and stroke (1). Nonetheless, current strategies for cardiovascular risk estimation (2) and reduction (3) appear inadequate. Both overt depression and subsyndromal depressive symptoms are currently recognized as independent cardiovascular risk factors comparable with smoking, obesity, hypertension, dyslipidemia, and diabetes (4–6). Depression is often associated with PsA (7) but the effective contribution of mood disorders in the cardiovascular burden of PsA patients is currently unknown.

Endothelial dysfunction (ED) is the inability of an artery to dilate in response to chemical or physical stimuli due to a reduced nitric oxide (NO) availability. ED plays a key role in early atherosclerosis and has the power to predict CVEs such as stroke, myocardial infarction, and cardiovascular death (8) consistently with the antiatherogenic and plaque-stabilizing properties of endothelium-derived NO (9). The ultrasound assessment of flow-mediated dilation (FMD) is a widely used method for assessing the endothelial function and strongly correlates with more invasive measures of the vasomotor responses (10).

Reduced FMD has been reported in both PsA patients (11) and depressed subjects (12) and related mainly to traditional cardiovascular risk factors and systemic inflammation. A dysregulated production of cytokines—particularly interleukin-6 (IL-6), tumor necrosis factor-α (TNF-α), and interleukin-17 (IL-17)—could be proposed as a unifying common feature between PsA, depression, and ED, as it plays a key role in the pathogenesis of psoriatic diseases and seems to be associated to mood disorders and impaired endothelial function in non-psoriatic cohorts (13, 14). Alternative or complementary mechanisms including heightened sympathetic arousal with excessive circulating catecholamines, abnormal neurohormonal function, reduced circulating endothelial progenitor cell, abnormal platelet activation, or increased oxidative stress could represent further mechanisms of impairment of vasomotor responses in PsA-depressed patients (15).

The aim of this study is therefore to evaluate the relationship of ED evaluated by FMD with depressive symptoms and serum cytokines in a cohort of PsA patients.

## Materials and Methods

### Study Design and Rheumatological Assessment

The study has a comparative cross-sectional design. The protocol has been approved by the Ethics Committee of Fondazione Policlinico Universitario A. Gemelli-IRCCS (FPG), Rome (Protocol n.0014580/18), and written informed consent was obtained from all participants. Consecutive patients, aged between 30 and 75 years who met classification criteria for psoriatic arthritis (CASPAR) (16) and on stable treatment for at least 3 months were enrolled at the outpatient rheumatology clinic of FPG between October 2018 and March 2019. PsA patients with diabetes, stroke, peripheral arterial disease, or overt coronary heart disease for previous myocardial infarction, coronary bypass surgery or angioplasty, coronary stenosis on angiogram, or evidence of exercise-induced myocardial ischemia, were excluded. Other exclusion criteria were history of neoplasm in the last 5 years, kidney or liver failure, chronic liver infection, drug or alcohol abuse, secondary hypertension, and treatment with antidepressant or corticosteroids.

Duration of PsA, history of peripheral arthritis, axial disease, dactylitis, enthesitis, skin disease and nail disease, pain intensity and patient global assessment (PtGA) on a 10-cm visual analog scale, erythrocyte sedimentation rate (ESR), and C-reactive protein (CRP) values were registered. Systemic, cutaneous, and articular examination—including tender and swollen joint count—was performed for all patients. Disease Activity in Psoriatic Arthritis (DAPSA) index and Psoriasis Area Severity Index (PASI) were calculated to assess disease activity. The use of conventional and biologic disease-modifying antirheumatic drugs (DMARDs) was noted.

### Assessment of Traditional Cardiovascular Risk Factors

Smoking habit, premature coronary artery disease in first- and second-degree relatives (male aged <55 years and female aged <65 years) and sedentary lifestyle were recorded. Body mass index (BMI) and waist-to-hip ratio (WHR) were recorded as well as proportion of patients with obesity (BMI ≥30 kg/m^2^) and abdominal obesity (WHR ≥0.94 for males and WHR ≥0.80 for females) were reported. Baseline resting blood pressure was measured by auscultatory technique, recording the average of three consecutive measures. Total cholesterol, high- (HDL) and low-density (LDL) lipoprotein cholesterol, and triglycerides were assessed before the visit according to standard practice, and atherogenic index of plasma (AIP) and LDL/HDL cholesterol ratio were computed. High-risk lipid profile has been defined as AIP >0.5 or LDL/HDL cholesterol ratio >3.5 in men and >3.0 in women (17). Ongoing treatment for systemic arterial hypertension and dyslipidemia were recorded (18).

Framingham risk score (FRS) (19) was used to estimate the 10 year risk of coronary heart disease death, non-fatal myocardial infarction, coronary insufficiency or angina, fatal or non-fatal ischemic or hemorrhagic stroke, transient ischemic attack, intermittent claudication, and heart failure for each patient according to traditional risk factors. The score was based on the following variables: age, sex, smoking status, total cholesterol, HDL cholesterol, and systolic blood pressure. This prediction model was adjusted for patients with chronic arthritis by a 1.5 multiplier (aFRS) (20). Notably, the age between 30 and 75 years was chosen as inclusion criteria, since FRS is validated in this range.

### Assessment of Depressive Symptoms

The presence of depression and the severity of psychological symptoms were evaluated by an experienced psychologist blinded to the other clinical data using the depression subscale of the Hospital Anxiety and Depression Scale (HDS). This scale was chosen because the items do not include somatic symptoms that may be caused by immunosuppressive drugs and extra-articular physical manifestations of PsA. Although it is not a basis for the clinical diagnosis of depression, a validated cutoff of 8 was used to define patients with significative depressive symptoms, who were referred to as depressed in this paper (21).

### Evaluation of Flow-Mediated Dilation

FMD was evaluated within 2 days from the screening visit by an experienced angiologist blinded to other clinical data. All participants were studied in the morning beginning between 8:00 and 9:00 am, fasting, and having avoided alcohol for at least 24 h and caffeine for at least 12 h. Vasoactive and non-steroidal anti-inflammatory medications were withheld for 48 h before the test. Subjects rested supine in a quiet, temperature-controlled environment for 20 min before the exam. The right brachial artery was imaged in the longitudinal plane 2 to 15 cm proximal to the antecubital fossa using a 17–5 MHz linear array transducer connected to an iU22 ultrasound machine (Philips Medical Systems, Monza, Italy). Depth and gain were selected to enable optimal identification of the anterior and posterior intimal interface between lumen and vessel wall on 2D grayscale images. Baseline images were then acquired. Blood flow was measured from the pulsed wave Doppler signal with a 60° insonation angle. After recording baseline values, a sphygmomanometer cuff was applied around the forearm, inflated to 250 mmHg, and left in place for 5 min, causing forearm ischemia and consequent dilation of downstream resistance vessels. Blood flow was measured over the first 15 s after cuff deflation, whereas arterial images were acquired between 60 and 90 s after cuff release (22).

### Assessment of Serum Cytokines

Blood samples for soluble biomarkers assays were collected from all enrolled PsA patients soon after FMD evaluation. Sera were collected from blood samples after centrifugation at 3,500 rpm for 15 min and stored at −80°C until the time of analysis. Serum levels of IL-6 were measured by ELISA (R&D Systems, Abingdon, UK) with a sensitivity of the test of 0.7 pg/ml. Serum levels of TNF-α and IL-17A were simultaneously measured by Luminex assay (R&D Systems, Minneapolis, USA) on a Luminex xMAP system (Bio-Plex 200 System, Bio-Rad Laboratories, Hercules, CA). Results were analyzed using a dedicated Bio-Plex Manager software and are expressed in picograms per milliliter.

### Statistical Analysis and Sample Size Calculation

Data were analyzed using IBM SPSS Statistics v26.0 (Armonk, NY, USA). Categorical variables were reported as numbers and percentages. Continuous variables were reported as mean ± SD or median and interquartile range (IQR), according to the distribution of the data. Normality of continuous variables was assessed by inspection of Q–Q (quantile-quantile) plots. Variables without Gaussian distribution were normalized using the logarithm transformation, when effective. Linearity of the relationship between continuous variables and the presence of significant outliers were assessed by scatterplot. The relationship between FMD and HDS or other paired continuous variables was explored using Pearson's (ρ) or Spearman's coefficient (*r*_s_) as indicated. The relationship between FMD and natural dichotomous variables was tested by point-biserial correlation (ρ) after ruling out the presence of significant outliers by boxplot and checking the homogeneity of variances by Levene's test. Hierarchical multivariate linear analyses were done with the percentage of FMD as the dependent variable. Variables showing a correlation with FMD were included in the model as predictors. The first block included predictors of FMD shared with general population (traditional cardiovascular risk factors, baseline brachial diameter), in the second block the model was adjusted for potential PsA-related predictors, in the last block HDS was added to the model. HDS was added in the last step in order to estimate the percentage of the total variance of FMD explained by depressive symptoms after controlling for potential confounding variables. Statistical significance of *F* modification was reported for each step. Independence of residuals was assessed by Durbin-Watson test, multicollinearity was excluded for ρ <0.700 for each couple of predictors. Homoscedasticity and normality of residual distribution were checked by visual inspection of a plot of standardized residuals vs. standardized predicted values and a probability plot, respectively. All tests were two sided. Statistical significance was defined as *p* < 0.05.

Consistent with the available literature, we calculated the minimum sample size based on the occurrence rate of depression (defined by HDS ≥8) of 33% in PsA patients defined by HDS ≥8, and an expected FMD value of 6.5 ± 3.0 in patients with depressed symptoms and 8.5 ± 3.0 in the other PsA patients. The α was set as double-sided 0.05 (5% level of significance), and β was set as 0.2 (90% power). Thus, we set the sample size as *n* = 50 in this study.

## Results

### Clinical Characteristics of PsA Patients, Serum Cytokines, Traditional Risk Factors for CVEs, and Depressive Symptoms

Fifty consecutive PsA patients were enrolled; the clinical characteristics of the study cohort are summarized in Table 1 and modifiable and non-modifiable risk factors for cardiovascular diseases in Table 2. The patients were treated according to standard of care at the time of the evaluation. Thirty-three patients (46.0%) were treated with conventional synthetic (cs)-DMARDs and specifically 19 with methotrexate, 16 with sulphasalazine, and two with leflunomide. Twenty-two patients (44.0%) were treated with biologic (b)-DMARDs, of whom five in monotherapy. In particular, 18 patients assumed an anti-TNFα, two patients secukinumab, and two ustekinumab. One patient was treated with apremilast.

**Table 1:** Clinical features of the enrolled PsA patients.

	**All patients**	**HDS ≥ 8**	**HDS <8**	***p*-Value**
*N*	50	20	30	–
Disease duration (years, mean ± SD)	5.9 ± 3.3	6.4 ± 3.5	5.6 ± 3.2	0.378
Age of onset (years, mean ± SD)	41.1 ± 11.9	40.4 ± 12.1	41.6 ± 12.0	0.732
Peripheral arthritis [*n* (%)]	50 (100.0)	20 (100.0)	30 (100.0)	–
Dactylitis [*n* (%)]	25 (50.0)	11 (55.0)	14 (46.7)	0.564
Enthesitis [*n* (%)]	28 (56.0)	11 (55.0)	17 (56.7)	0.907
Spondylitis [*n* (%)]	11 (22.0)	3 (15.0)	8 (26.7)	0.269
Psoriatic skin disease [*n* (%)]	40 (80.0)	15 (75.0)	25 (83.3)	0.470
Psoriatic nail disease [*n* (%)]	20 (40.0)	10 (50.0)	10 (33.3)	0.239
TJC on 68 joints [median (IQR)]	1.0 (0.0–4.5)	1.5 (0.0–8.8)	1.0 (0.0–2.5)	0.138
SJC on 66 joints [median (IQR)]	0.0 (0.0–2.0)	1.0 (0.0–3.5)	0.0 (0.0–2.0)	0.222
Pain intensity on VAS (cm, mean ± SD)	5.1 ± 2.7	6.2 ± 2.6	4.1 ± 2.8	0.008
PtGA (cm, mean ± SD)	3.6 ± 2.3	4.7 ± 2.3	2.9 ± 2.0	0.004
DAPSA (mean ± SD)	14.0 ± 9.4	18.4 ± 10.5	11.1 ± 7.6	0.007
PASI [median (IQR)]	0.5 (0.0–3.5)	0.6 (0.0–4.2)	0.5 (0.0–3.5)	0.935
HAQ-DI [median (IQR)]	0.889 (0.000–1.120)	0.935 (0.370–1.778)	0.060 (0.000–0.870)	0.001
Conventional DMARDs (%)	32 (64.0)	13 (65.0)	19 (63.3)	0.904
Biologic DMARDs and apremilast [*n* (%)]	23 (46.0)	8 (40.0)	15 (50.0)	0.487
No DMARDs [*n* (%)]	13 (26.0)	5 (25.0)	8 (26.7)	0.895
IL-6 [pg/ml, median (IQR)]	0.21 (0.01–2.08)	0.82 (0.01–2.56)	0.02 (0.01–1.42)	0.165
TNF-α [pg/ml, median (IQR)]	3.52 (2.23–5.57)	3.88 (2.56–5.63)	3.36 (2.22–5.09)	0.442
IL-17A [pg/ml, median (IQR)]	0.01 (0.01–0.24)	0.01 (0.01–0.11)	0.01 (0.01–0.35)	0.760

*PsA, psoriatic arthritis; HDS, depression subscale of the Hospital Anxiety and Depression Scale; SD, standard deviation; TJC tender joint count; SJC, swollen joint count; IQR, interquartile range; VAS, visual analog scale; PtGA, patient global assessment; DAPSA, disease activity in psoriatic arthritis; HDA, high disease activity; MDA, moderate disease activity; LDA, low disease activity; PASI, psoriasis area severity index; IL, interleukin; TNF, tumor necrosis factor; DMARDs, disease-modifying antirheumatic drugs*.

**Table 2:** Traditional risk factors for cardiovascular disease.

	**All patients**	**HDS ≥ 8**	**HDS <8**	***p*-Value**
*N*	50	20	30	–
Age (years, mean ± SD)	50.7 ± 10.2	51.5 ± 11.1	50.1 ± 9.6	0.638
Male sex [*n* (%)]	21 (42.0)	4 (20.0)	17 (56.7)	0.010
Family history of premature CVD [*n* (%)]	13 (26.0)	5 (25.0)	8 (26.7)	0.895
BMI (kg/m^2^, mean ± SD)	26.0 ± 4.0	24.7 ± 3.8	26.8 ± 4.0	0.067
Obesity [*n* (%)]	10 (20.0)	3 (15.0)	7 (23.3)	0.365
WHR (mean ± SD)	0.88 ± 0.14	0.92 ± 0.10	0.86 ± 0.16	0.162
Abdominal obesity [*n* (%)]	34 (68.0)	14 (70.0)	20 (66.7)	0.804
Current smokers [*n* (%)]	13 (26.0)	6 (30.0)	7 (23.3)	0.599
Sedentary lifestyle [*n* (%)]	32 (64.0)	12 (60.0)	20 (66.7)	0.630
SBP at the time of the study (mmHg, mean ± SD)	123 ± 16	123 ± 15	123 ± 17	0.818
DBP at the time of the study (mmHg, mean ± SD)	80 ± 11	80 ± 10	81 ± 11	0.815
Antihypertensive treatment [*n* (%)]	18 (36.0)	8 (40.0)	10 (33.3)	0.630
Total cholesterol (mg/dl, mean ± SD)	207 ± 31	207 ± 33	207 ± 31	0.994
HDL-cholesterol (mg/dl, mean ± SD)	63 ± 18	64 ± 22	61 ± 16	0.615
LDL-cholesterol (mg/dl, mean ± SD)	123 ± 33	125 ± 32	122 ± 35	0.783
Triglycerides (mg/dl, mean ± SD)	112 ± 75	101 ± 46	118 ± 87	0.454
Atherogenic index of plasma [median (IQR)]	0.44 (−0.09 to 0.96)	0.49 (−0.10 to 1.00)	0.42 (−0.06 to 0.96)	0.740
Atherogenic index of plasma at risk [*n* (%)]	24 (48.9)	10 (50.0)	14 (46.7)	0.912
LDL/HDL cholesterol ratio (mean ± SD)	2.17 ± 0.97	2.16 ± 0.85	2.18 ± 1.05	0.930
LDL/HDL cholesterol ratio at risk [*n* (%)]	8 (16.0)	3 (15.0)	5 (16.7)	0.599
Lipid-lowering treatment [*n* (%)]	8 (16.0)	3 (15.0)	5 (16.7)	0.599
aFRS [%, median (IQR range)]	10.7 (7.2–14.5)	11.3 (7.2–18.2)	10.8 (8.0–15.2)	0.501

*HDS, depression subscale of the Hospital Anxiety and Depression Scale; SD, standard deviation; CVD, cardiovascular disease; BMI, body mass index; WHR, waist-to-hip ratio; SBP, systolic blood pressure; DBP, diastolic blood pressure; HDL, high-density lipoproteins; LDL, low-density lipoprotein; IQR, interquartile range*.

Four patients (8.0%) were in high disease activity, 19 (38.0%) in moderate disease activity, 20 (40.0%) in low disease activity and 7 (14.0%) in remission according to DAPSA score while under treatment. Twenty (40.0%) patients were depressed according to HDS ≥8 with an average HDS of 6.8 ±3 0.3. Depressed and non-depressed patients did not significantly differ according to disease features and serum levels of IL-6, TNF-α, and IL-17A (Table 1).

The median 10 year risk of CVEs according to aFRS was 10.7% (IQR, 7.2–14.5%). Depressed patients were less frequently male (20.0% vs. 56.4%, *p* = 0.01) while they did not differ according to the other recorded cardiovascular risk factors (Table 2).

### Flow Mediated Dilatation

Single correlations of FMD and HDS with traditional risk factors for CVD and other clinical features are shown in Supplementary Table 1. Baseline brachial artery diameter on ultrasound was 3.7 ± 0.7 mm and mean FMD was 7.9% ± 3.6%. There was a statistically significant, negative correlation between endothelial function according to FMD and severity of depressive symptoms according to HDS (ρ = −0.339, *p* = 0.016), explaining 11% of the variation in endothelium-dependent vasodilation. The relationship was similar if the cutoff of HDS ≥8 was considered to define depressed patients (ρ = −0.322, *p* = 0.022). Regarding traditional risk factors for CVD, there was a statistically significant, strong negative correlation between FMD and age (ρ = −0.507, *p* = 0.001) and a moderate negative correlation with aFRS (*r*_s_ = −0.453, *p* < 0.001) and Log(aFRS) (ρ = −0.423, *p* = 0.002). Of notice, aFRS was normalized by log transformation because of the markedly skewed distribution of the data.

**Figure 1:**
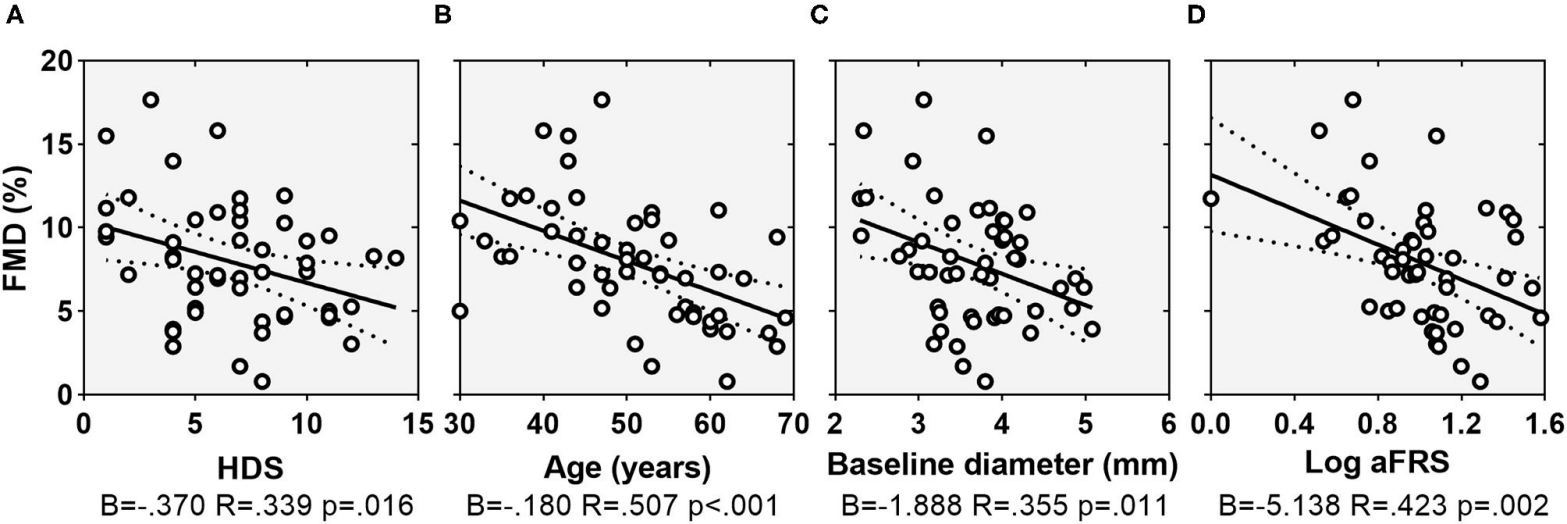
Scatterplots showing the relationship between FMD and HDS **(A)**, age **(B)**, baseline arterial diameter **(C)**, and Log(aFRS) **(D)**. Dotted lines indicate 95% confidence interval. FMD, flow-mediated dilation; HDS, depression subscale of the Hospital Anxiety and Depression Scale; aFRS, adjusted Framingham risk score.

**Figure 2:**
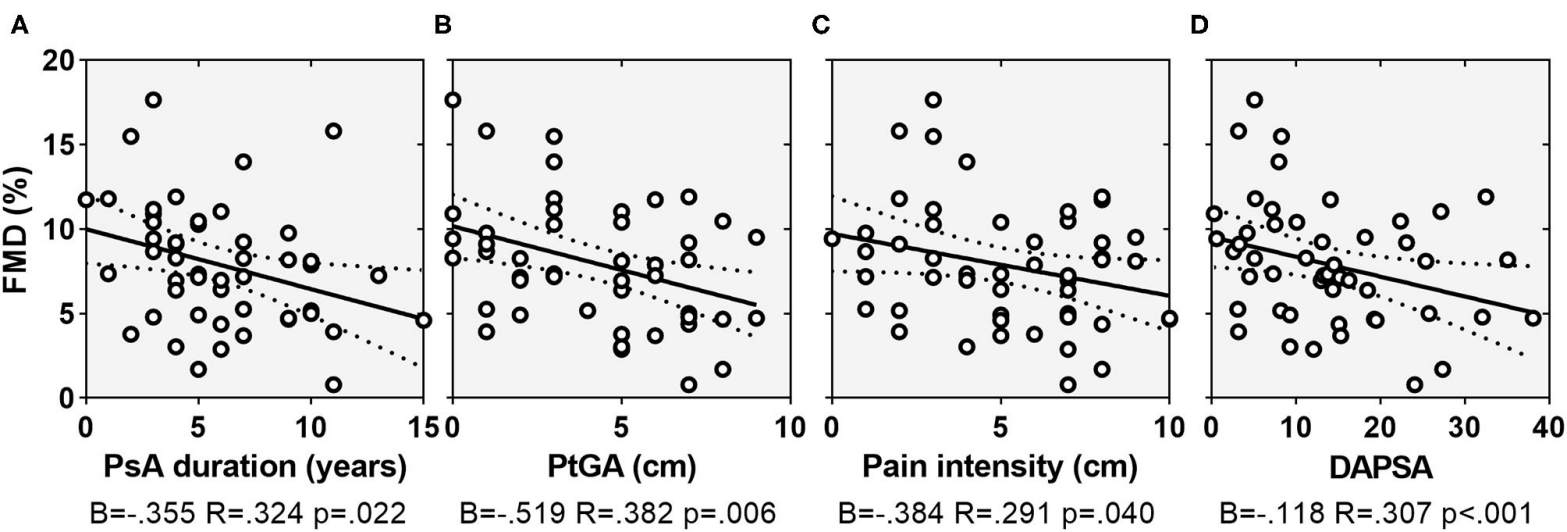
Scatterplots showing the relationship between FMD and PsA duration **(A)**, PtGA **(B)**, pain intensity on VAS **(C)**, and DAPSA **(D)**. Dotted lines indicate 95% confidence interval. FMD, flow-mediated dilation; PsA, psoriatic arthritis; PtGA, patient global assessment; VAS, visual analog scale; DAPSA, disease activity in psoriatic arthritis.

There was a statistically significant correlation between gender and HDS (ρ = −0.320, *p* = 0.024), with females showing higher depressive scores compared with males (7.7 ± 2.8 vs. 5.5 ± 3.6). As concerns disease characteristics, FMD correlated with PsA disease duration (ρ = −0.507, *p* = 0.001), intensity of pain (ρ = −0.507, *p* = 0.001), and DAPSA (ρ = −0.507, *p* = 0.001) while HDS correlated with TJC on 68 joints (*r*_s_ = 0.312, *p* = 0.028), the intensity of pain (ρ = 0.483, *p* < 0.001) and DAPSA (ρ = 0.495, *p* < 0.001) (Figures 1, 2). Notably, there was not a significant correlation between FMD or HDS and serum cytokine concentrations and classic acute-phase reactants.

Multiple regression analyses was carried out to assess the relative contribution of the variables in predicting FMD. Predictors were chosen according to both theoretical importance and statistical significance on bivariate correlation. Since pain intensity and PtGA were both highly correlated with DAPSA (ρ = 0.821, *p* < 0.001 and ρ = 0.791, *p* < 0.001, respectively) and each other (ρ = 0.802, *p* < 0.001), these measures have not been entered simultaneously in the model, to avoid multicollinearity. We decided to use pain intensity in the model since it was included in DAPSA formula and because psychological factors have an established role in the experience of pain. Therefore, hierarchical regression was run to predict FMD, entering as variables age, baseline arterial diameter, and Log(aFRS) in the first step. PsA duration and pain intensity were added in the second step and HDS in the third step. Regression coefficient (*R*^2^)values are reported in Table 3. Conventional cardiovascular risk factors (age, aFRS) and baseline arterial diameter accounted for 31.0% of the total FMD (*F* = 6.88, *p* < 0.001) with age independently associated with the endothelial function (β = −0.425, *p* = 0.008). PsA-related variables in the second step, the explained variance increased up to 40.8% (*F* = 3.64, *p* = 0.034) and pain intensity (β = −0.370, *p* = 0.015) and age (β = −0.271, *p* = 0.026) were both significantly associated with the endothelial function in the adjusted model. The inclusion of HDS in the third step contributed to an additional 6.4% of the variance in predicting FMD (*F* = 5.21, *p* = 0.027). It should be noted that at the last step the correlation between FMD and HDS remained significant on multivariate regression analysis (β = −0.297, *p* = 0.027) suggesting an independent effect of the depressed mood on endothelial function. Age was also significantly associated with FMD (β = −0.372, *p* = 0.011) in the full-enter model.

**Table 3:** Hierarchical linear regression analysis of FMD in patients with PsA.

	**Predictors**	***B***	**CI 95%**		***p* (*B*)**	**β**	***F* change**	***p* (*F* change)**	***R*^**2**^**	***R*^**2**^ change**
Step 1	Age	−0.152	−0.261	−0.042	0.008	−0.425	6.88	0.001	0.310	–
	Baseline artery diameter	−1.159	−2.837	0.519	0.171	−0.218				
	Log(aFRS)	−0.482	−5.047	4.084	0.883	−0.040				
Step 2	Age	−0.132	−0.237	−0.025	0.015	−0.370	3.64	0.034	0.408	0.098
	Baseline artery diameter	−0.869	−2.522	0.783	0.295	−0.163				
	Log(aFRS)	−1.087	−5.440	3.266	0.617	−0.089				
	PsA duration	−1.016	−0.431	0.123	0.270	−0.140				
	Pain severity	−0.358	−0.670	−0.045	0.026	−0.271				
Step 3	Age	−0.132	−0.233	−0.032	0.011	−0.372	5.21	0.027	0.472	0.064
	Baseline artery diameter	−1.182	−2.785	0.422	0.145	−0.222				
	Log(aFRS)	−1.016	−5.177	3.146	0.625	−0.084				
	PsA duration	−0.098	−0.368	0.171	0.466	−0.090				
	Pain severity	−0.178	−0.516	0.161	0.295	−0.135				
	HDS	−0.324	−0.610	−0.038	0.027	−0.297				

*FMD, flow-mediated dilatation; PsA, psoriatic arthritis; CI, confidence interval; aFRS, adjusted Framingham risk score; PsA, psoriatic arthritis; HDS, depression subscale of the Hospital Anxiety and Depression Scale*.

## Discussion

In the present study, we examined the endothelial function assessed by FMD according to depressed mood and key serum cytokines in a clinically well-characterized cohort of PsA patients. Our main observation was an inverse correlation between endothelial function and severity of depressive symptoms according to HDS, confirmed after adjusting for other relevant variables such as age, baseline brachial diameter, traditional cardiovascular risk factors (aFRS), disease duration, and pain severity. In the multivariable linear model, the largest variation of FMD was explained by age and aFRS, as reported in the general non-psoriatic population (23, 24), while disease duration and pain severity were the only disease characteristics that were related to FMD, explaining further 9.8% of its variance. Expanding the hierarchical model, we found that the variation of FMD independently explained by HRS was of 6.4%. Even if this percentage may appear small or clinically irrelevant, it must be emphasized that very small differences in FMD can predict major CVEs (8).

These data allow us to speculate that the gap between predicted and actual incidence of CVEs could be in part explained by a quote of endothelial dysfunction related to an eventual concurrent depressive status. In this perspective, the investigation of depressive symptoms in PsA could contribute to a more accurate stratification of cardiovascular risk, since mood disorders are often under-recognized and under-treated in these patients (25). It should also be noted that mood disorders have a stronger impact on cardiovascular disease burden in women than in men (26) and that depressive symptoms are more frequent in female PsA patients (27). The identification of depressive symptoms may therefore be crucial in female PsA patients that could be mistakenly considered at low risk in premenopausal age. Notably, the relationship between depressive symptoms and FMD showed a linear trend, suggesting that evaluating the depressive status as dichotomous phenomenon (i.e., depressed vs. non-depressed) may eventually be inadequate in the stratification of cardiovascular risk.

**Figure 3:**
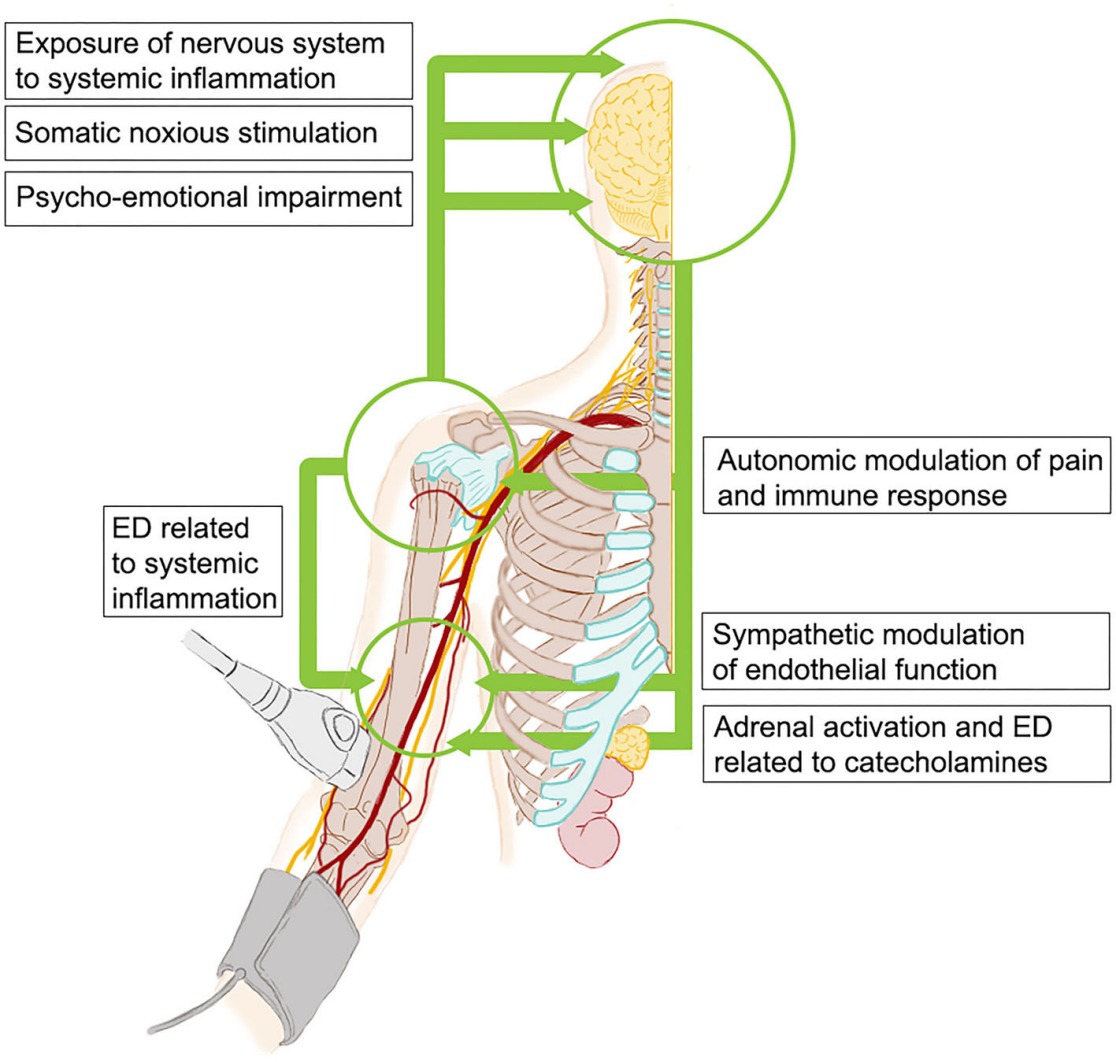
Potential mechanisms of interconnections between neurohormonal system, inflammation, and endothelium in patients with PsA and depressive symptoms. PsA, psoriatic arthritis; ED, endothelial dysfunction.

Mechanisms of ED in PsA or depression are still unclear, but they are expected to be multiple and overlapping. The crosstalk between central and peripheral nervous system, the inflammatory and autoimmune response, and the cardiovascular system is complex (Figure 3), and our current understanding of these interactions is incomplete, particularly in PsA patients. It is well-known that, even if traditional cardiovascular risk factors are frequently reported in PsA and depression, ED and cardiovascular risk are independent of them in both cases (28–30). Systemic inflammation is considered a major actor in ED in PsA and other chronic inflammatory diseases (13), indeed we failed in detecting a correlation between endothelial function and acute-phase reactants or inflammatory molecules (i.e., IL-6, TNF-α, and IL-17A), consistently with previous reports on FMD in PsA patients (11, 28, 30). Interestingly, ED has been related to the cumulative rather than the transient exposure to inflammation (30, 31), and we consistently found an inverse correlation between FMD and disease duration. Depression itself has also been associated with a sustained state of systemic inflammation and increased concentrations of inflammatory molecules in patients without overt systemic inflammatory diseases (14) and, in this regard, we previously reported that depressive symptoms were independently associated with elevated IL-6 in a larger cohort of unselected PsA patients (32). An alternative or complementary explanation is that other non-inflammatory mechanisms may interfere with endothelial function in these patients. These include deregulation of the hypothalamic-pituitary-adrenal axis with elevated circulating cortisol levels (33, 34) and an imbalance between the sympathetic and parasympathetic systems (35, 36). Such mechanisms might be heightened in PsA-depressed patients. Coherently with this hypothesis, autonomic dysfunction has been reported in PsA patients (37), particularly in association with psychoemotional impairment (38). Notably, we also fund that ED was inversely correlated with pain, that is cross-linked with sympathetic activation (39).

The limitations of the present study include the lack of a control group and the cross-sectional design with the consequent inadequacy to more strongly support the relationship between endothelial dysfunction and depressive symptoms. Moreover, we did not directly measure potential non-inflammatory determinants of ED nor the cumulative exposure to inflammation or depressive mood (40). Lastly, we did not assess the impact of different immunosuppressants on endothelial function since the study was not powered for this aim.

The study has some strengths, too. To the best of our knowledge, this is the first study assessing the relationship between ED and depressive symptoms in patients with PsA. The patients have been thoroughly studied in terms of traditional cardiovascular risk factors, and we applied stringent exclusion criteria in order to limit confounding factors. Patients treated with antidepressants, for example, were excluded from the study because of the effect of antidepressant drugs on endothelial function (41).

## Conclusions

In our cohort of PsA patients, depressive symptoms were related to ED. If validated in longitudinal studies, this evidence would encourage a systematic research of depressive symptoms as a part of a correct assessment of cardiovascular risk in PsA, helping to raise the effectiveness of prevention strategies. The future research agenda should clarify if the choice of both immunosuppressant and antidepressant treatment in PsA could be personalized according to effects on endothelium, as part of cardiovascular prevention strategies.

## Data Availability Statement

The original contributions presented in the study are included in the article/Supplementary Material, further inquiries can be directed to the corresponding author/s.

## Ethics Statement

The protocol and the template informed consent forms were reviewed and approved by Committee on Research Ethics of Catholic University of the Sacred Heart. The patients/participants provided their written informed consent to participate in this study.

## Author Contributions

All authors gave substantial contributions to the conception or design of the work, acquisition, analysis, or interpretation of data, drafting the work or revising it critically for important intellectual content, and final approval of the version published.

## Conflict of Interest

The authors declare that the research was conducted in the absence of any commercial or financial relationships that could be construed as a potential conflict of interest.

## Publisher's Note

All claims expressed in this article are solely those of the authors and do not necessarily represent those of their affiliated organizations, or those of the publisher, the editors and the reviewers. Any product that may be evaluated in this article, or claim that may be made by its manufacturer, is not guaranteed or endorsed by the publisher.
