# Supplementary material for: Depression and Endothelial Dysfunction in Psoriatic Arthritis: Is There Any Possible Relationship?
Source: Front Med (Lausanne). 2021 Aug 27;8:669397. doi: 10.3389/fmed.2021.669397 (PMC8429609; doi:10.3389/fmed.2021.669397)
Supplement: Supplementary file 1 [file Table_1.DOCX]

| **Supplementary Table 1 - Correlations of FMD and HDS with traditional risk factors for CVD, clinical features and serum cytokines** | | | | | | |
| --- | --- | --- | --- | --- | --- | --- |
|  | **FMD** | | | **HDS** | | |
|  | **ρ** | **r_s_** | ***P*** | **ρ** | **r_s_** | ***P*** |
| HDS | **-.339** |  | **.016** | - | - | - |
| HDS≥8 | **-.322** |  | **.022** | - | - | - |
| Age | **-.507** |  | **<.001** | .007 |  | .960 |
| Male sex | .121 |  | .403 | **-.320** |  | **.024** |
| Family history of premature CVD | -.087 |  | .548 | -.054 |  | .711 |
| BMI | .005 |  | .971 | -.159 |  | .269 |
| WHR | -.097 |  | .503 | .173 |  | .228 |
| Current smoke | .049 |  | .734 | .029 |  | .839 |
| Sedentary lifestyle | -.008 |  | .975 | .059 |  | .683 |
| SBP at the time of the study | .092 |  | .527 | -.014 |  | .921 |
| DBP at the time of the study | .074 |  | .610 | -.072 |  | .621 |
| Antihypertensive | -.085 |  | .557 | .581 |  | -.040 |
| Total cholesterol | -.109 |  | .466 | -.040 |  | .789 |
| HDL-cholesterol | -.091 |  | .550 | .135 |  | .371 |
| LDL-cholesterol | -.033 |  | .830 | -.046 |  | .762 |
| Triglycerides | -.018 |  | .903 | -.064 |  | .675 |
| Glucose | -.212 |  | .167 | -.017 |  | .915 |
| LDL/HDL cholesterol ratio | .100 |  | .513 | -.117 |  | .444 |
| Atherogenic index of plasma |  | -.015 | .922 |  | -.064 | -625 |
| Lipid lowering drug | -.234 |  | .102 | .015 |  | .916 |
| aFRS |  | **-.453** | **.001** |  | -.110 | .447 |
| Log(aFRS) | **-.423** |  | **.002** | -.094 |  | .517 |
| Duration of PsA | **-.374** |  | **.008** | .128 |  | -375 |
| Age of onset of PsA | -.221 |  | .123 | -.123 |  | .394 |
| Dactylitis | -.052 |  | .717 | .007 |  | .962 |
| Enthesitis | -.020 |  | .893 | -.090 |  | .535 |
| Spondylitis | -.105 |  | .466 | -.082 |  | .570 |
| Psoriatic skin disease | -.083 |  | .566 | -.131 |  | .366 |
| Psoriatic nail disease | .181 |  | .208 | .104 |  | .473 |
| CRP |  | -.113 | .434 |  | -.044 | .760 |
| Log(CRP) | -.092 |  | .526 | -.046 |  | .752 |
| ESR |  | -.086 | .555 |  | .065 | .655 |
| Log(ESR) | -.075 |  | .604 | .013 |  | .929 |
| TJC from 68 joints |  | -.175 | .225 |  | **.312** | **.028** |
| SJC from 66 joints |  | -.204 | .156 |  | .218 | .129 |
| Pain intensity on VAS | **-.291** |  | **.040** | **.483** |  | **<.001** |
| PtGA | **-.382** |  | **.006** | **.444** |  | **.001** |
| DAPSA | **-.307** |  | **.030** | **.495** |  | **<.001** |
| PASI |  | -.091 | .530 |  | -.030 | .834 |
| cDMARDs | .109 |  | .450 | -.080 |  | .581 |
| bDMARDs | -.153 |  | .289 | -.213 |  | .137 |
| No DMARDs | -.131 |  | .364 | .113 |  | .436 |
| IL-6 |  | -.184 | .200 |  | .118 | .414 |
| TNF-α |  | -.129 | .373 |  | .101 | .484 |
| IL-17A |  | .139 | .336 |  | -.085 | .556 |
| *Bold indicates variables with p<.005.*  *Abbreviations: FMD flow-mediated dilatation, CVD cardiovascular disease, HDS depression subscale of the hospital anxiety and depression scale, ρ Pearson's or point biserial correlation coefficient, rs Spearman’s coefficient, BMI body mass index, WHR waist to hip ratio, SBP systolic blood pressure, DBP diastolic blood pressure, HDL high-density lipoproteins, LDL low density lipoprotein, aFRS adjusted Framingham risk score, PsA psoriatic arthritis, CRP C-reactive protein, ESR erythrocyte sedimentation rate, TJC tender joint count, SJC swollen joint count, VAS visual analogue scale, PtGA patient global assessment, DAPSA disease activity in psoriatic arthritis, PASI psoriasis area severity index, DMARDs disease-modifying antirheumatic drugs, IL interleukin, TNF tumour necrosis factor.* | | | | | | |
